# Feasibility study of the Home-based Exercises for Responsible Sex (HERS) intervention to promote correct and consistent condom use among young women

**DOI:** 10.1186/s40814-021-00885-1

**Published:** 2021-07-27

**Authors:** Nicola Knights, Nicole Stone, Tom Nadarzynski, Katherine Brown, Katie Newby, Cynthia A. Graham

**Affiliations:** 1 Centre for Sexual Health Research, Department of Psychology, University of Southampton, Southampton, UK; 2 School of Social Sciences, University of Westminster, London, UK; 3 School of Life and Medical Sciences, University of Hertfordshire, Hatfield, UK

**Keywords:** Condom, Condom use, Intervention

## Abstract

**Background:**

Male condoms are effective in preventing common sexually transmitted infections (STIs) and unintended pregnancy, if used correctly and consistently. However, condom use errors and problems are common and young people report negative experiences, such as reduced pleasure. The Kinsey Institute Home-Based Exercises for Responsible Sex (KIHERS) is a novel condom promotion intervention for young women, which aims to reduce condom errors and problems, increase self-efficacy and improve attitudes towards condoms, using a pleasure-focussed approach. The study objective was to test the operability, viability and acceptability of an adapted version of the KIHERS intervention with young women aged 16–25 years in the United Kingdom (UK) (Home-Based Exercises for Responsible Sex-UK (HERS-UK).

**Methods:**

A repeated-measures single-arm design was used, with a baseline (T1) and two follow-up assessments (T2 and T3), conducted 4 weeks and 8 weeks post intervention over a 3-month period. Participants were provided a condom kit containing different condoms and lubricants and were asked to experiment with condoms alone using a dildo and/or with a sexual partner. Ten process evaluation interviews were conducted post intervention.

**Results:**

Fifty-five young women received the intervention; 36 (65%) completed T2 and 33 (60%) completed T3. Condom use errors and problems decreased, self-efficacy increased and attitudes towards condoms improved significantly. The proportion of participants who reported using a condom for intercourse in the past 4 weeks increased from T1 (20; 47%) to T2 (27; 87%) and T3 (23; 77%) and using lubricant with a condom for intercourse increased from T1 (6; 30%) to T2 (13; 48%)) and T3 (16; 70%). However, motivation to use condoms did not change. Cronbach’s alpha scores indicated good internal consistency of measures used. Qualitative data provided strong evidence for the acceptability of the intervention.

**Conclusions:**

HERS-UK was implemented as intended and the recruitment strategy was successful within a college/university setting. This feasibility study provided an early indication of the potential effectiveness and acceptability of the intervention, and the benefits of using a pleasure-focussed approach with young women. Measures used captured change in outcome variables and were deemed fit for purpose. Future research should explore cost-effectiveness of this intervention, in a large-scale controlled trial using a diverse sample and targeting young women most at risk of STIs.

**Supplementary Information:**

The online version contains supplementary material available at 10.1186/s40814-021-00885-1.

## Key messages regarding feasibility

- This is the first feasibility study conducted of the Home-based Exercises for Responsible Sex (HERS-UK) intervention to promote correct and consistent condom use among young women aged 16*–*25 years in the UK.
- Key findings included strong evidence for the acceptability of the intervention to young women, the viability of recruitment methods and outcomes measures used, and initial evidence that the intervention might change attitudes towards condoms and increase condom-related skills and self-efficacy.
- The findings suggest that a larger scale randomised controlled trial of HERS could be conducted with few, if any, adaptations required.

## Background

Sexually transmitted infections (STI) are a serious public health problem and have been increasing in recent years. Public Health England (PHE) reported almost half a million diagnoses of STIs in 2019, a 5% increase from 2018, the most common being chlamydia and gonorrhoea, although diagnoses of syphilis have also increased [1]. In fact, in young women, diagnoses of both gonorrhoea and syphilis increased by 31%. Young people aged 15–24 years are at higher risk of contracting STIs due to more frequent changes of partner [1]. Compared with young men, young women face more serious complications from STIs, such as pelvic inflammatory disease, which can increase the risk for ectopic pregnancy and infertility [2]. Young women are also at risk of unplanned pregnancies and a large-scale survey in the UK found approximately 45% of pregnancies were unplanned in women aged 16-19 years [3].

Male condoms can be highly protective against the transmission of STIs [4], but incorrect and/or inconsistent condom use with new or casual sex partners is an important factor underlying the prevalence of STIs among young people [5]. However, almost half of young people did not use a condom when having sex with a new partner [6]. PHE launched a campaign in 2017 to reduce rates of STIs in young people, by raising awareness of the potentially serious consequences of STIs and normalising condom use [6].

The protection condoms provide against STIs is also reduced when they are not used from the start to the finish of sex (termed ‘incomplete use’), which is more likely to occur among couples who experience problems whilst using condoms or dislike using them [7, 8]. Negative attitudes and problems with ‘fit and feel’ can stop young people from using condoms [9, 10]. Sexual health interventions which have tried to increase condom use by focusing on improving men’s condom use skills and knowledge have had only limited success [11].

More recently, interventions to improve effective condom use have promoted pleasure and fun when using condoms and lubricants [12, 13]. The Kinsey Institute Homework Intervention Strategy® (KIHIS) is a brief behaviour change, condom promotion intervention developed to improve condom skills, self-efficacy and enjoyment in young men. The impetus for behaviour change comes from encouraging solitary practice of using condoms in a ‘no pressure’ situation (without a partner) with different brands and sizes of condoms, and focussing on pleasurable sensations whilst experimenting. Evidence for initial effectiveness has been demonstrated in two pilot studies (one involving heterosexual men and one involving men who have sex with men) conducted in the USA and Canada. These studies reported a reduction in condom problems and improved self-efficacy, skills and experiences when using condoms [12, 13].

The KIHIS was adapted for use in the UK (HIS-UK), and a feasibility trial was recently completed [14]. Young men were provided with a face-to-face training session on how to apply a condom correctly and a variety of condoms and lubricants to experiment with at home. The aim was to find a condom that was right for them, and increase their focus on pleasurable sensations — thereby challenging beliefs that condoms reduce pleasure. The intervention was well-received by both young men and health care professionals, and results from post-intervention questionnaires indicated that there was an increase in lubricant use, reduction in errors and problems, and increased motivation to use condoms.

### Barriers to condom use in young women

Although under-researched compared with men’s experiences, women also report problems and dissatisfaction with condoms, such as discomfort, dryness and reduced sensation [15, 16]. In addition, women often apply condoms to their male partners and report errors and problems when doing so [15]. Educating men on how to apply condoms correctly and how to make condom use more pleasurable is an important public health strategy. However, very few interventions to date have focussed on this approach with women. Interventions have tended to focus more on gender power dynamics, such as condom negotiation skills [17], and have targeted subgroups of women who are at high risk for contracting human immunodeficiency virus (HIV)/STIs such as sex workers and African American women [18, 19].

The affective attitude that condoms reduce sexual pleasure and enjoyment, and provoke disgust or dislike, is associated with condom non-use for men and women [10, 20–22]. In fact, studies have found that both men and women report unprotected sex as being more pleasurable [10]. Sexual pleasure is an important consideration for women when choosing a contraceptive method [16–19] and barrier methods that interfere with the enjoyment of sex are less likely to be appealing.

Condoms can not only reduce sensation and pleasure and cause vaginal irritation for women, but can also interfere with perceived closeness to a partner and prove to be an interruption to sex or ‘spoil the mood’ [23–27]. In addition, condoms can cause arousal loss and interfere with orgasm, resulting in their discontinuation [28, 29] and the smell of latex can be a ‘turn off’ for some women [16]. Therefore, it is just as important for women as for men that they have the opportunity to find a condom that they can enjoy using, so safe sex can also be pleasurable sex [29].

There is evidence to suggest that interventions incorporating a pleasure-focussed approach, such as those that encourage positive associations with condoms, are more effective in improving attitudes and increasing condom use than those that focus on instilling fear and accentuating risk [30–34]. There has also been a move to include pleasure as part of sex education to enhance the relevance for young people, especially women, whose pleasure has been largely ignored and to challenge the view that safety and pleasure are mutually exclusive [35–37].

Women also apply condoms to their male partners and are just as likely as men to make errors and experience problems when using them [38, 39], which can affect both their own and their partners’ experience. Common errors reported by young women include putting the condom on the wrong way around, using a condom that had dried out, not using lubricant, and rushed application, which can cause problems such as slippage and breakage, erection loss and discomfort [40]. Experiencing problems that affect sexual experience may lead to negative attitudes towards condoms and discontinuation. It is therefore important that interventions address condom use errors and problems in both men and women to improve the condom use experience [5].

Not only lack of objective skill, but also lack of *perceived* skill or confidence may be a barrier to young women using condoms, especially if their partner is unsure about how to use condoms correctly. Self-efficacy is the belief and confidence that one is capable of successfully performing a given behaviour [41]. Global condom use self-efficacy (i.e. perceived ability to buy, carry, negotiate and use a condom correctly) and other relevant self-efficacy dimensions (such as using a condom under the influence of substances, or when highly aroused) are associated with intention to use condoms in young women [42] and can predict actual and intended condom use [43, 44]. However, high condom use self-efficacy does not always ensure correct and consistent condom use. For example, an individual may believe that they are using a condom correctly even if they are making consistent errors; therefore, interventions should target actual condom use skills [45].

The KIHIS was adapted for women and the KIHERS created by the Condom Use Research Team (KI-CURT) at the Kinsey Institute. The aim of the intervention is to improve women’s experience with condoms and their ability to use condoms and lubricants as part of mutually pleasurable and safe sexual activity. The intervention incorporates core training to increase condom skills and self-efficacy, with home-based practice using a range of different condoms and lubricants. Young women are asked to rate the condoms and lubricants practised with, on various dimensions, including how pleasurable the experience was. A recent pilot study of KIHERS in the USA found that the intervention was acceptable to young women and that condom use errors and problems decreased, whilst condom-related attitudes and self-efficacy improved. Condom experiences were also more positively rated post intervention [46].

### Underlying theoretical framework

It is important that behaviour change interventions have a solid theoretical framework. The KIHERS intervention incorporated the information-motivation-behavioural skills (IMB) model, [47, 48] which is an empirically validated and comprehensive health behaviour change framework, developed to reduce sexual risk behaviours. The model contains three constructs required to affect behaviour change — information, motivation and behavioural skills. Firstly, providing information helps the individual decide whether to engage in a particular health behaviour. Personal motivation involves addressing attitudes that may influence the individual’s likelihood of engaging in that behaviour, and social motivation involves increasing social support, i.e. normalisation of the behaviour within a social group. Finally, behavioural skills include perceived and objective skills as well as confidence (i.e. self-efficacy) for carrying out the behaviour. The IMB model has a sound theoretical basis for modifying sexual risk behaviour [49–52] and interventions using an IMB framework have had a significant impact on changing behaviour in young people [53, 54].

The objective of the current study was to adapt the KIHERS into a culturally and health care-appropriate intervention for delivery to young women (aged 16–25 years) within community settings in the UK (HERS-UK), and to test it for viability, operability and acceptability in readiness for a full trial.

## Methods

### Design

The research included a *consultation and design* work package to refine and finalise the content of HERS-UK. This phase involved conducting three focus groups and four individual interviews with young women who provided feedback and suggestions on all aspects of the intervention, and input from an advisory group of sexual health experts who provided further advice and guidance with regard to the intervention content and delivery. In addition, a *feasibility* work package was included to test recruitment and retention strategies, intervention delivery and adherence to the protocol, data collection and assessment procedures, appropriateness of proposed outcome measures and acceptability of the intervention to young women.

The feasibility study included intervention delivery and process evaluation using quantitative questionnaire data and qualitative interview data. Ethical approval was obtained from the University of Southampton ethics committee.

For the main feasibility study, a repeated-measures, single-arm design was used, with participants providing baseline and two sets of follow-up data via an online survey. Participants provided baseline data (T1), after which they met with the researcher to receive the KIHERS core training. Participants were then given 3 weeks to test and try out a range of condoms and lubricants and a second survey was administered 4 weeks after the end of the testing period. The final survey (T3) was administered 4 weeks later. Questionnaire and demographic data were collected using a secure online platform called Lifeguide which is a University of Southampton developed tool that has been used extensively in web-based interventions. The programme incorporated a text/email messaging service, so participants received reminders when it was time to complete follow-ups and condom ratings.

All young women had the opportunity to provide feedback about their experience of study participation after completing T3. Ten young women additionally took part in process evaluation interviews and were asked about their experiences of the intervention. Interviews were audio-recorded and transcribed by hand by the first author. A thematic analysis was conducted by the first author, using Braun and Clarke’s methodology [55]. Transcripts were coded using an inductive approach, whereby themes were generated from the data and no pre-defined coding framework used. Identified themes were discussed and finalised with the study PI.

### Recruitment

The aim was to recruit approximately 50 young women to take part in the main feasibility study. The initial strategy was to recruit young women from local youth organisations, but many of the organisations approached were overwhelmed with supporting service users with mental health problems and did not feel they could currently engage with a sexual health intervention. An alternative approach of recruiting from colleges and universities in Hampshire was more successful. The researcher provided an educational session on condom use and an introduction to the study to students, which was arranged with either the school nurse or the assistant head teacher. After the session, young women received a variety of free condoms and lubes and posters containing links to the study website, where prospective participants could download an information sheet and register for the study. Inclusion criteria were (1) any gender identity (with attributes of a biological woman, i.e. a vagina); (2) aged 16–25; (3) does not always use a condom with sexual partners; (4) dislike condoms or experience problems when using them. Participants were excluded if they had a penis or a latex allergy.

Over 150 young women registered for the study, and 55 were selected to participate on a first come, first served basis — participants were contacted in order of registration and those who responded were booked in for core training. When the number of participants who had completed the baseline survey and received core training reached 55, recruitment was halted. Recruitment began in July 2019 and data collection ended in January 2020.

### Intervention content and delivery

Prior to registration, participants were able to address any queries or concerns with the researcher before agreeing to take part. Participants gave online consent before beginning the survey. Baseline (T1) data comprised information on demographics and participants’ sexual activity and condom use, including the impact of condoms on sexual experience, errors and problems when using condoms, attitude towards condoms, and confidence in using and applying condoms.

Participants were then contacted by the researcher to arrange a time to meet to receive the core training session, which lasted approximately 50 min. The intervention was delivered using a motivational interviewing (MI) approach that can support and enable behaviour change [56] by the first author (who was trained in MI). MI has been successful in enhancing motivation for behaviour change in adolescents and young people with a range of problems that include addiction and sexual risk-taking [57–59] and has demonstrated effectiveness as part of brief behaviour change interventions for young people [60, 61]. The purpose of the session was to provide information about condoms and lubricants, with an emphasis on maximising pleasure and reducing discomfort and interruption to improve the sexual experience, and thereby change attitudes towards condoms. Information was provided about condom and lube use within the context of vaginal, anal and oral sex. Participants were also informed about different sizes of condoms, and provided with a size guide (see online supplementary file), as many young women in the consultation and design phase had mentioned that size was a problem for them and their partners.

The second aim of the core training session was to develop skills in using condoms correctly, thus reducing errors and problems and increasing self-efficacy, and positive condom attitudes and experiences. The researcher demonstrated how to apply a condom correctly using a realistic size dildo, and young women had the opportunity to practise themselves, and receive feedback. An instruction and information leaflet was also provided with instructions on how to apply a condom correctly, so young women could refer to this when practising at home. In addition, the leaflet contained information about different types of condoms and lubes, tips on how to make condom use more pleasurable, and condom testing and rating instructions, so participants were clear about what they were being asked to do. Participants were also told about novel ways of applying condoms (such as by mouth) in the session.

Along with the instruction and information leaflet, participants received a condom kit to take home. The condom kit was designed with input from the young women that had contributed to the consultation and design phase. They provided suggestions for all aspects of the intervention, including the attributes of the different condoms, the packaging of the kit, the study logo (see online supplementary file), the colour and size of the dildo (realistic-sized, and pink or purple) and the inclusion of a condom holder, so that condoms could be carried discreetly. Young women had also expressed a preference for having the option to rate condoms tested, both alone, and with a partner.

The kit contained 10 different condoms (two of each type, 20 in total) designed for women’s pleasure and comfort, such as textured, flavoured, coloured, extra sensitive, non-latex and coated with stimulating gel. In addition, a condom designed for women, with a clean smell and attractive packaging was included, and one that came in fun, unique packaging. The researcher discussed the different attributes of each condom with the participant. In addition, a selection of water and silicone-based lubes were included and a realistic-sized dildo (available from https://www.annsummers.com/sex-toys/sex-toys-dildos/5inch-realistic-jelly-dildo/118146.html). Each kit contained two of each type of condom (20 in total), so that young women could experiment with the condom alone using the dildo, and/or with a sexual partner.

### Follow-up assessments after completion of the core session

After the session, participants had 3 weeks to experiment with the condoms and lubes at home. After trying out each individual condom, they were asked to rate each one on attributes such as smell, texture and packaging and overall, how enjoyable it had been to use, e.g. ‘I liked the texture of the condom material.’ Responses were scored on a 5-point scale from ‘strongly disagree’ to ‘strongly agree’. Higher scores indicated a more positive experience of using a particular condom.

Four weeks after rating the condoms, participants received a text/email reminder to complete T2 and then 4 weeks after, the final follow-up (T3). T2 and T3 questions asked about sexual activity in the previous 4 weeks and all the same questions as T1, with the exception of demographic information. After completing T2, participants received a gift pack of condoms and lubes, which was either delivered to them at their school/college or posted discreetly to their home address. After completion of T3, participants received £25 cash for their time. The ten participants who completed post-intervention process interviews received an additional £10.

### Outcome measures

The objective of HERS-UK was to improve the sexual experience of using condoms for young women, address negative attitudes towards condoms, increase self-efficacy and skill to use condoms and ultimately, increase motivation to use condoms. Below we describe the measures used to assess these variables.

#### Demographic questions

Demographic questions included age, ethnicity, highest level of qualification, employment status, postcode, sexual orientation, relationship status, number of lifetime sexual partners and previous condom use (see Table 1 for demographic characteristics of the sample).

**Table 1:** Demographic characteristics (*N* = 55)

	% (*N*)
Ethnicity	
White British	85.5 (47)
White European	3.6 (2)
Asian British	3.6 (2)
Black British	3.6 (2)
Mixed	1.8 (1)
Other	1.8 (1)
Relationship status	
Romantic relationship with one partner	67.3 (37)
Frequent or occasional casual sex	32.7 (18)
Sexual orientation	
Heterosexual	82 (45)
Bisexual	18 (10)
Highest level of education	
GCSE or equivalent	56 (31)
A Level or equivalent	29 (16)
Degree or equivalent	6 (3)
Masters or equivalent	9 (5)

#### Effect of condoms on sexual experience

The degree to which using a condom affected arousal, desire, pleasure, orgasm and emotional closeness to partner was measured using items taken from the HERS-US pilot study [13]. A sample item was ‘The last time you had sex using a condom, to what degree did using a condom impact your arousal?’ Responses were made using a 5-point scale from ‘greatly decreased’ to ‘greatly increased’, with a higher score indicating a more positive impact. T1 Cronbach’s alpha was .88.

#### Condom use errors and problems

The 16-item Condom Use Errors/Problems Survey for Women (CUES-W) [62] assessed common errors and problems during penile-vaginal and penile-anal sex when using a condom. A sample item was ‘The last time you had sex, the condom broke.’ Responses were ‘yes’, ‘no’ or ‘don’t know’. An additional item was included that asked the participant if she had applied the condom to her sexual partner and if so, she was asked if she had applied it the right way around, squeezed the air out, and left a space at the tip. The total CUES-W score was the number of errors and problems reported.

#### Condom attitudes

Condom attitudes were measured using 13 items: 7 from the Effect on Sexual Experiences subscale of the Condom Barriers Scale (CBS) [63]. A sample item was ‘condoms don’t feel good’. An additional 6 items were included with a more positive slant, e.g. ‘condoms can make sex more pleasurable’, adapted from the Pleasure Associated with Condoms subscale of the Multidimensional Condom Attitudes Scale (MCAS) [64]. Responses were scored using a 5-point scale from ‘strongly agree’ to ‘strongly disagree’, with negatively slanted items reverse scored. A higher score indicated a more positive attitude towards condoms. T1 Cronbach’s alpha was .84.

#### Condom use self-efficacy

Subscales of the Condom Use Self-Efficacy Scale (CUSES) [65] were used that included Mechanics and Intoxicants, with a total of 16 items. Self-efficacy for negotiation was not included. CUSES includes items relating to self-efficacy for technical skills in using a condom, e.g. ‘I feel confident in my ability to use a condom correctly’; for minimizing disruption to the sexual experience, e.g. ‘I feel confident that I could apply a condom to my partner without breaking the mood’; when purchasing and carrying condoms, e.g. ‘I feel confident that I could purchase condoms without feeling embarrassed’ and when using substances, e.g. ‘I feel confident that I would remember to use a condom even after I have been drinking.’ Responses were scored on a 5-point scale from ‘strongly disagree’ to ‘strongly agree’, with negative items reverse scored. A higher score indicated greater self-efficacy. T1 Cronbach’s alpha was .91.

#### Reasons for using/not using condoms

Participants were provided with statements as to why they had or had not used a condom, e.g. ‘dislike using them’ and could tick as many as were applicable.

#### Motivation to use condoms

At the end of T1, T2 and T3, participants were asked to respond to the statement ‘I really want to use condoms with my partner.’ Responses were scored on a 5-point scale from ‘strongly agree’ to ‘strongly disagree’. A higher score indicated higher motivation.

#### Process evaluation questions

A subgroup of ten participants were asked about their experience of taking part in the intervention in a one-to-one face-to-face interview. Process interview questions covered the following topics: motivation for taking part in the study; previous experience of condoms; their experiences taking part in the study, including the introductory session with the researchers, rating the condoms, using the website, and completing the questionnaires, and what they thought about the kits.

#### Sample size

We did not do a power calculation to determine the sample size for the quantitative analyses but chose a target *N* of 50 because previous feasibility studies had used similar numbers of participants. Women who participated in the design and consultation phases were offered the opportunity to participate in the main feasibility study but only a small number did and the majority of participants were recruited from organizations and colleges.

### Quantitative data analysis

IBM SPSS Statistics 26 was used for all analyses. The objective of a feasibility study is primarily to test the acceptability of measures, but changes in scores across time points can indicate that measures are appropriate to capture any impact of an intervention, in readiness for a full trial. The inter-item reliability of the scale measures administered at baseline was tested, using Cronbach’s alpha. Binary logistic regression was used to predict the odds of dropping out of the study (yes/no) based on deprivation and level of education (GCSE/A Level or above). These two variables were chosen based on findings from a feasibility study on the HIS-UK intervention that these variables were related to the likelihood of study completion and compliance [14]. Deprivation was quantified by dichotomising categories of deprivation into ‘most’ and ‘least’ deprived, using the English Indices of Multiple Deprivation (2019) [66]. Outcome measures using questionnaire scores were compared across T1 (baseline) and T2 and T3 (post intervention) using the repeated-measures analysis function within the General Linear Model. Post hoc paired sample *t* tests were used if the overall model was significant. If violation of sphericity was present, then a Greenhouse-Geisser correction was applied and a non-parametric Wilcoxon signed-rank test used for post hoc analysis. A priori predictions were that any significant change in outcome variables was likely to occur between T1 and T2, with change being maintained at T3.

## Results

### Participants

All 55 participants were between the ages of 16 and 25 (*M* 18.40 years, SD 2.6) and the majority were students. Most participants lived in areas of higher socioeconomic status in line with the English Indices of Multiple Deprivation (2019) [66]. The number of lifetime sexual partners ranged from 1 to 50, with three being the median number reported. The lifetime prevalence of chlamydia diagnosis was 7.3 % (*N* = 4).

Participants were able to provide open text responses about their motivation to take part in the study. Responses were categorised as follows (from the most to least frequent): (1) how to make sex with a condom more pleasurable; (2) the opportunity to try out a variety of condoms and lubes; (3) to learn more about how to use condoms, and reduce problems such as splitting and dryness; (4) interest in and desire to gain more confidence generally with regard to female sexual health and pleasure; (5) to find an alternative method of contraception to hormonal methods.

### Condom ratings and adherence

Of the 55 participants who received the intervention, 73% (*N* = 40) went on to rate condoms. For each condom, participants had the opportunity to complete 20 ratings: 10 alone and 10 with a partner. The average number of ratings was 7.5 (range 1–16); 15 (38%) participants were fully adherent, rating all 10 condoms. More ratings were done with a partner (*N* = 189; 62%) vs. alone (*N* = 114; 38%) and the dildo was used in the majority of ‘alone’ ratings 103 (90%) vs. 11 (10%). Lubricant was used in a third of ratings (29%, *N* = 87) and 65 (75%) of participants who used lubricant reported that it increased pleasure. The mean time to complete ratings was 12.5 min (SD 9.1). The most highly rated condoms were the brand designed for women, with a clean smell and attractive packaging, the studded design with fun, uniquely designed packaging and the non-latex brand. The coloured and flavoured condoms received the lowest ratings, although young women said that flavoured condoms could make oral sex more fun.

### Retention

**Fig. 1:**
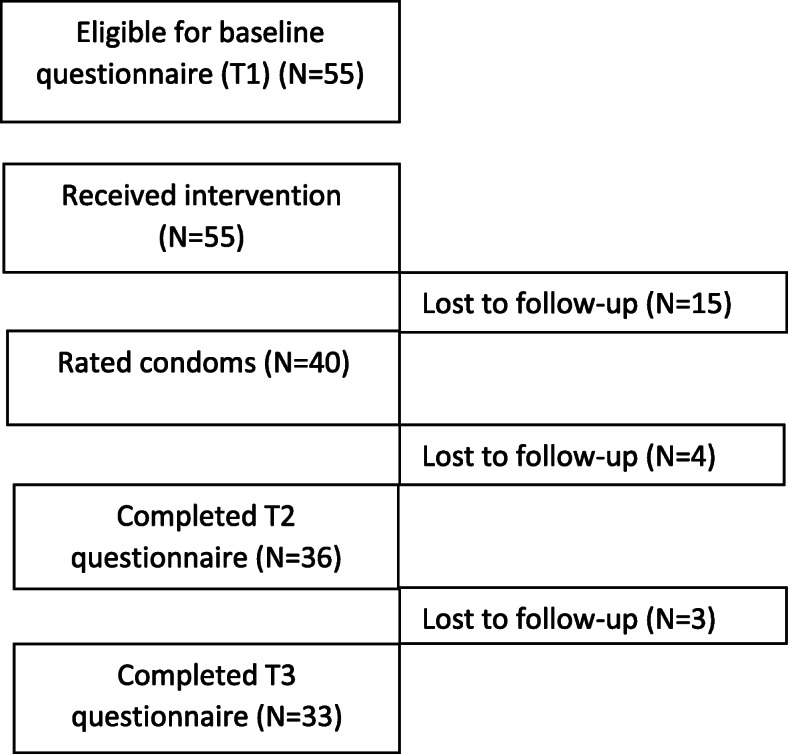
Summary of participant flow

After rating the condoms, 36 women (65%) completed T2 and 33 (60%) completed T3 (see Fig. 1). A logistic regression analysis predicted the likelihood of dropout based on the level of qualification and deprivation. The overall model was significant *X*^2^ (2) = 12.74, *p* = .002. Residing in areas of least deprivation (*p* = .05) and higher level of education (*p* = .009) significantly increased the odds of completing the study.

### Outcome assessment

Participants were asked why they had or had not used a condom when having sexual intercourse with a partner at T1, T2 and T3 in the past 4 weeks; they could tick as many reasons as applicable (see Table 2). The proportion of participants who reported using a condom for intercourse in the past 4 weeks increased from T1 (20; 47%) to T2 (27; 87%) and T3 (23; 77%) and using lubricant with a condom for intercourse increased from T1 (6; 30%) to T2 (13; 48%) and T3 (16; 70%).

**Table 2:** Reasons for condom use and non-use at T1, T2 and T3

Reason	T1	T2	T3
*Reason for not using*			
Dislike using them	27% (*N* = 15)	3% (*N* = 1)	9% (*N* = 3)
Using other contraceptive method	27% (*N* = 15)	8% (*N* = 3)	15% (*N* = 5)
No STIs	20% (*N* = 11)	5.5% (*N* = 2)	12% (*N* = 4)
Did not think about using condom	20% (*N* = 11)	0	0
Lack confidence using condoms	7% (*N* = 4)	0	0
*Reason for using*			
To avoid STIs/HIV	33% (*N* = 18)	33% (*N* = 12)	58% (*N* = 19)
To improve sex/increase fun	3.5% (*N* = 2)	47% (*N* = 17)	36% (*N* = 12)
To make sex last longer	9% (*N* = 5)	8% (*N* = 3)	15% (*N* = 5)
To avoid mess	13% (*N* = 7)	20% (*N* = 8)	36% (*N* = 12)
As a contraceptive method	27% (*N* = 15)	53% (*N* = 19)	51% (*N* = 17)

Participants who had used a condom in the past 4 weeks were asked at T1, T2 and T3 about errors and problems (CUES-W) (see Tables 3 and 4) and effects on sexual experience, using items from the HERS-US pilot study, which asked about the perceived impact of using a condom on desire, arousal, pleasure, closeness and orgasm. The most common errors at T1 were not checking the expiry date and not checking for damage, and the most common problems were discomfort and problems fitting the partner. There was an overall decrease in reported errors and problems from T1 (*M* 6, SD 3.20) to T2 (*M* 2.7, SD 1.8), *t* (13) = 3.28; *p* = .006, 95% CI [2.5-5], but no further significant reduction at T3 (*M* 2.7, SD 2.1). There was no significant change in the reported effect of condoms on sexual experience across time points.

**Table 3:** Percentage of participants reporting errors at each time point

	Did not check the expiry date	Did not check for damage	Used oil-based lube	Contact with a sharp object	Applied late	Removed during sex	Did not squeeze the air out	Did not leave space at the tip	Applied wrong way around
**T1**	62% (*n* = 18)	59% (*n* = 17)	3% (*n* = 1)	21% (*n* = 6)	31% (*n* = 9)	10% (*n* = 3)	71% (*n* = 12)	35% (*n* = 6)	35% (*n* = 6)
**T2**	62% (*n* = 15)	50% (*n* = 12)	4% (*n* = 1)	12% (*n* = 3)	33% (*n* = 8)	12% (*n* = 3)	10% (*n* = 2)	10% (*n* = 2)	5% (*n* = 1)
**T3**	45% (*n* = 10)	36% (*n* = 8)	*n* = 0	9% (*n* = 2)	27% (*n* = 6)	22% (*n* = 5)	20% (*n* = 4)	45% (*n* = 9)	15% (*n* = 3)

**Table 4:** Percentage of participants reporting problems at each time point

	Problem fitting partner	Uncomfortable	Partner lost erection when applying	Partner lost erection when having sex	Condom broke	Condom slipped off during sex	Condom slipped off as partner pulled out
**T1**	48% (*n* = 14)	76% (*n* = 22)	41% (*n* = 12)	34% (*n* = 10)	21% (*n* = 6)	21% (*n* = 6)	38% (*n* = 11)
**T2**	37% (*n* = 9)	33% (*n* = 8)	16% (*n* = 4)	20% (*n* = 5)	12% (*n* = 3)	4% (*n* = 1)	16% (*n* = 4)
**T3**	18% (*n* = 4)	22% (*n* = 5)	13.5% (*n* = 3)	18% (*n* = 4)	4.5% (*n* = 1)	4.5% (*n* = 1)	13.5% (*n* = 3)

All participants were asked about their attitude towards condoms (using items from the CBS/MCAS) and self-efficacy for condom use (CUSES) at each time point. Attitude improved from T1 (*M* 31.6, SD 6.1) to T2 (*M* 37.6, SD 6.6) *Z* = 4.3, *p* < 0.001, 95% CI [34.2–38.1] but no further significant change occurred at T3 (*M* 39.3, SD 7.1). Self-efficacy increased from T1 (*M* 52, SD 11.4) to T2 (*M* 59.6, SD 8.5) Z = 3.82, *p* = < 0.001, 95% CI [54.7–60.4] but no further significant change occurred at T3 (*M* 61.1, SD 7.9). For both those in established relationships and those with casual partners, motivation to use condoms did not change.

For a summary of the change in outcome measures across T1–T3, see Table 5.

**Table 5:** Change in outcome measures across T1, T2 and T3

Outcome measure	T1	T2	T3	Significance of change
Condom use errors and problems (*M*)	6	2.7	2.7	a*F*(2, 18) = 8.6, *p* = 0.002
Effect on sexual experience (*M*)	11.8	15.2	14.4	a*F*(2, 18) = 2.4, *p* = 0.113
Attitude towards condoms (*M*)	31.6	37.6	39.3	b*F*(1.7, 54.4) = 27.55, *p* < 0.001
Self-efficacy for condom use (*M*)	52	59.6	61.1	b*F*(1.6, 50.1) = 22.22, *p* < 0.001
Motivation to use condoms (*M*)	3.5	3.4	3.6	n/s

a = ANOVA with repeated measures

b = ANOVA with repeated measures, Greenhouse-Geisser correction applied

### Process evaluation interviews

The thematic analysis conducted on the ten process evaluation interviews identified six themes, described below.

#### Theme 1: Fun and relaxed vs. clinical and ‘intimidating’

Young women felt that sexual health interventions could be intimidating for some, especially if they were too ‘clinical’ or ‘in your face’. However, participants said that they felt comfortable being part of the HERS-UK Condom Study as it was informal, relaxed and fun. Sexual health interventions that focussed on condoms and the risk of STIs and those that were too sexually explicit were unappealing. Most young women wanted condoms that had female-friendly packaging and were ‘discreet’. The use of language was important to make young women feel comfortable; for example, the use of the word ‘exploration’ or ‘experimentation’ was preferred as opposed to ‘masturbation’. Some young women were more sexually confident and comfortable using more explicit words, but still felt that it was better to use neutral language initially, and see what different individuals felt comfortable with. One of the aims of the feasibility study had been to adapt KIHERS for use with young women in the UK, so making sure the intervention contained culturally appropriate language was an important consideration.

*“Website and logo didn’t make you nervous to be a part of the study… it was not too serious, it looked relaxed and laid back - not intimidating’….”*

*“The study was fun… and the packaging not dull and boring.”*

No significant embarrassment or psychological discomfort was reported, but one participant felt that care needed to be taken when asking questions about casual sex, as this might infer judgement. This was especially relevant for women who traditionally are encouraged to enjoy sex as part of a loving relationship, with casual sex associated with risk and fear of contracting STIs.

“*I think there should be an option to differentiate between ‘dating’ sort of casual sex and having casual sex with someone you've just met.”*

#### Theme 2: Pleasure and empowerment

Despite the acknowledged sensitivity around women and sexual pleasure, participants welcomed the emphasis on female pleasure and being in the study had left some feeling ‘empowered’. There was felt to be a stigma around women enjoying sex and young women suggested that studies such as HERS-UK could help to address this. For some, it was the first time that they had been explicitly asked to focus on, and consider, their own pleasure.

*“It’s quite nice to be asked about your experience, rather than just how it feels for the man – empowering….”*

Rating condoms on their own for some women provided an opportunity to focus on their own bodies and their own enjoyment and not having to ‘rush’ to accommodate their partner’s ‘readiness’ or pleasure. Some young women had previously talked about the application of the condom as signifying the end of foreplay, which may not have corresponded with when they were ready to begin intercourse.

*“Advantage of rating alone, you could relax and focus on your own pleasure… take your time….”*

In addition, some young women had found it useful to try out a condom alone to ‘get to know it’, which increased their confidence before using it with a casual partner. It could be intimidating for those who were less sexually experienced to apply and use a condom with someone that they did not know that well. Those with steady partners thought it was a good idea to find a condom that felt good for them, and could enhance sexual pleasure when using it with their partner.

*“Trying out condoms on the dildo is good for girls with more casual partners, as you don’t want to get out a new condom with someone that you don’t know anything about.”*

*“I enjoyed using the dildo… it gave me a good chance to engage with the condom before trying it out with my partner….”*

#### Theme 3: Not ‘one size fits all’

What young women said they benefitted from most about being in the study was the opportunity to try out a variety of condoms to find one that suited them. Most were limited by which condoms were available for free, which was not always suitable; there was a particular problem cited with size variation. Cost was a significant barrier to being able to experiment with different condoms. For most young women, their experience of using free condoms had been mainly negative, which had put them off using condoms altogether.

*“They give you Pasante and nothing else… you don’t get lube, you have to get that yourself…if you wanted to try a few different condoms and lubes, it would cost you about £50 upwards.”*

Ratings provided the opportunity for young women to explore different attributes of a condom — such as fragrance, texture, colour — and focus on what they liked and what they did not like because it made them evaluate a condom in a way they had never done before.

*“The rating forces you to think about the condom in more depth….”*

*“I didn’t know what was important to me… now I do – heat transmission and thickness.”*

The pack had included condoms designed to enhance women’s pleasure and these had been well received overall, but condom preference was still a very personal thing. For example, some women reported increased pleasure and sensation when using ribbed and dotted, but for others, they caused discomfort.

“*Very good experience with this condom for me and my partner. I think the dotted texture heightened stimulation and made the sex more pleasurable.”*

*“I felt that the studs on the condom were too big and caused a little pain and irritation.”*

Some young women commented that there should have been more size variation in the pack, as when they were rating with a partner they encountered problems with condoms being too tight, or slipping off.

*“We tried this condom but unfortunately it was far too tight that we couldn’t get it over half way without him being in pain.”*

#### Theme 4: Sharing the experience — physical and emotional closeness

Although most young women found the dildo useful and enjoyed using it, there was acknowledgement that it felt different to a penis. Young women felt it was better to have the option to rate with both the dildo and a partner, if possible, to ‘get the best of both worlds’. For example, testing with a partner provided a more ‘natural’ physical sensation. However, it was not just the physical sensation that was important when rating an experience with a partner, but the emotional closeness — being able to share the experience and have fun together.

*“I preferred testing condoms with my boyfriend because of heat transduction…it feels better, more natural.”*

*“I think it’s good to experience both sides of it if you can – rating alone and with a partner…I made a lot of jokes about it with my partner….”*

In addition, for those with regular partners, testing the condoms together facilitated discussion about what they both enjoyed when using condoms. For some, these kinds of conversations had not happened before participating in the study.

*“The best thing was when we laid them all out and decided what we wanted to try….I also asked him - ‘what did you think of it?’ – we did talk about it more.”*

#### Theme 5: Identifying problems, finding solutions

Prior to completing the intervention, most young women had accepted condom use problems such as slippage, breakage, and discomfort as largely unsolvable and ‘normal’ — an intrinsic part of using a condom — which is likely to have contributed to negative attitudes. They discussed problems they had encountered while using condoms previously, and how being in the study had helped them to actively address these. Most felt that the study was very ‘hands-on’, meaning that they were not just passive participants, but actively engaging with the kit, and experimenting with the various condoms and lubes to improve their experience. For example, dryness had caused discomfort for many young women, but using lubes had alleviated some of the discomfort.

*“With the lubes, it’s much more helpful, because whereas before we couldn’t finish… now we can.”*

*“I mixed together the water and silicone lube to get the right consistency.”*

For some young women, condoms had been an interruption to sex, but learning how to apply them skilfully had improved this to some degree; experimenting with different techniques such as mouth application was also seen as a good thing, as it increased options for those who felt that condoms spoiled the mood.

*“Nice to know how to apply a condom myself, as it’s then easier to incorporate it into foreplay… I think now it’s less of an interruption than it was….”*

Many young women reported problems with using the wrong size condoms and some had previously been unaware of the different sizes available.

*“Trying different sizes helped as I previously struggled with this with my partner and didn’t know about the different size options.”*

#### Theme 6: Functional and fun

Most young women had seen condoms in purely functional terms prior to participating in the study: ‘a necessary evil’ and associated with STIs. Condoms were not generally associated with fun or pleasure. Certainly, the sex education that most young women had received had only talked about condoms in terms of STIs or unintended pregnancy and had done nothing to address their reputation as ‘pleasure destroyer’. However, having the opportunity to experience different colours, shapes, textures, flavours etc. had challenged this perception. For some young women, this was the first time that they had been able to experiment with a variety of condoms. Some had experimented with ribbed and dotted condoms prior to being in the study, but most had been unaware of the different sizes and other attributes of condoms and had never really considered how condoms might enhance women’s pleasure.

*“Condoms are more fun now… they are not just a thing to stop getting pregnant….”*

*“I definitely learned a lot more about the different types of condoms and how they can be more pleasurable for women.”*

## Discussion

### Feasibility findings

HERS-UK was implemented as intended and no major adaptations from the original KIHERS intervention were required. The intervention was straightforward to deliver, and the one-to-one core training session with the researcher worked well in a variety of locations that suited participants. All participants received the core components of the training session: basic instruction on how to apply a condom correctly; introduction to the selection of condoms and lubes; discussion with the researcher about their own problems/experiences with condoms and possible solutions. Sessions were individualised to some degree, depending on the experiences and preferences of individual young women. For example, some participants had more experience applying condoms or a greater degree of discomfort/dislike in using them, and in these cases, the researcher would spend more time on these topics.

Using a MI approach to deliver the intervention was valuable, as this approach emphasises respect, autonomy and empathy and supports self-efficacy. In addition, behavioural change occurs because of its relevance to the young person’s own values. An MI approach explores and addresses ambivalence for change, i.e. ‘I want to use condoms to protect myself from STIs, but they make sex less pleasurable’, which was a common dilemma for young women in the study. Participants found the opportunity to discuss problems they had experienced with condoms and to receive a ‘menu’ of possible solutions very useful, in keeping with the MI approach. Reducing condom use problems, and thus improving the condom experience, is likely to increase self-efficacy and also improve attitudes. It was clear from the qualitative process interviews, and talking to young women in the core training session, that problems with condoms, such as breakage and discomfort, were viewed as just a normal part of condom use, and something that had to be ‘put up with’, rather than addressed, which is likely to have contributed to negative attitudes.

Participants reported differing experiences of sex education and often had not been taught explicitly about condom application. Teaching young women how to use condoms correctly in a relaxed ‘women-only’ setting and also providing the opportunity to practise alone, with a dildo, is likely to increase practical skill and self-efficacy. Young women in our study found that having the opportunity to try out individual condoms before using them with partners was very useful.

Providing detailed information about condoms, especially with regard to size, was also a good strategy for improving self-efficacy. Many young women had struggled with condom size issues and had experienced problems with condoms breaking or slipping off, which had decreased their confidence and contributed to negative attitudes. In fact, although overall participants were very positive about the condom kit, one criticism was that there was not enough size variation. This is something that should be addressed in any future trial, especially as young women are likely to want to experiment with partners.

Participants engaged very well with HERS-UK and expressed satisfaction with the text/email reminders and did not find them intrusive. Most dropouts occurred before the rating period, which indicates that some young women may have just wanted to receive the condom kit and not intended to engage with the study. Dropout from rating to T3 was low; generally, those who rated condoms continued with the intervention until T3. Interestingly, two of the women who dropped out after rating condoms were both participants with no partnered sexual experience, but they had wanted to learn more about condoms and lubricants before having sex for the first time. There was evidence to suggest that dropout was more likely to occur in those living in less affluent areas, and with lower levels of education. In subsequent studies, it would be worth investing more time in understanding how the intervention could be adapted to increase retention rates for those young women.

Nearly three-quarters of those who received the kit provided condom ratings. In fact, young women engaged well with the rating component of the intervention, with an average of 7.5 ratings per participant, and an average rating time of 12.5 min per practice session. Participants also wrote thoughtful comments about the condoms they were rating. Experimentation with condoms and lubricants was acceptable and a fun part of the intervention for most of the participants — both alone and with a partner. Participants who rated condoms after experimenting alone reported that they enjoyed using the dildo, although the option to rate with a partner was also important and more ratings were completed with a partner than alone. Some young women preferred the more ‘natural’ feel of trying out condoms with a partner and some felt that it was more fun to do the intervention with another person. For those who had regular partners, some enjoyed the experience of choosing a condom that suited them both.

The rating component of the intervention was important in that it explicitly focussed attention on the various attributes of condoms and pleasurable sensations. Negative attitudes are likely to remain unchallenged if young women do not have the opportunity to re-evaluate their experiences and feelings towards condoms. Young women were also appreciative of the focus on finding condoms and lubricants that suited them as an individual, as they had seldom had the opportunity to find a condom that they were comfortable using. The focus was usually on finding a condom that suited the male partner.

Process evaluation interviews provided further support for the acceptability of HERS-UK; the emphasis on female pleasure was especially well received. Younger participants appeared to be equally comfortable with HERS-UK as older participants or those with greater sexual experience. HERS-UK was designed for young women who are having/or have had sexual intercourse and experienced problems with or disliked using condoms but it seems feasible that it could be adapted for those who have yet to begin their sexual lives.

Although teachers and staff in colleges/universities were not interviewed, the intervention appeared to be acceptable to this group, who supported recruitment and were keen to receive outside expertise in sexual health for their students. However, it was more difficult to recruit from youth organisations, where staff were dealing with youth with mental health problems and considered sexual health to be a lower priority. The recruitment strategy worked very well in schools and colleges, although this did require a significant investment of time and building relationships. Over 150 students registered for the study — most after receiving an introductory talk and free condoms and lubricant samples. However, some participants had registered after seeing the study poster and had not attended a presentation, so this could be a feasible alternative recruitment strategy. Providing free samples of different condoms and lubricants is a relatively cost-effective way of engaging and retaining participants.

### Limitations

Some study limitations need to be acknowledged. There was limited diversity in terms of ethnicity, but this was more likely a representation of the local population from where participants were drawn. Further work could be done on adapting the intervention (if required) for those young women from Black, Asian and Minority Ethnic backgrounds. For example, in cultures where it is less acceptable for women to talk about sexual pleasure, some aspects of the wording in recruitment could be adapted. There might also be a requirement to add a negotiation component to the intervention for those young women from backgrounds where there is a significant gender power imbalance. In addition, although the study included bisexual young women, more work could be done on making HERS-UK accessible and relevant for individuals with diverse sexual orientation and gender identities.

Another potential limitation was that participants were recruited solely from colleges and universities, as it was not possible to work with local youth organisations who were dealing with large volumes of mental health issues in young people. Therefore, those from the most disadvantaged backgrounds may not have been adequately represented in our sample. In fact, there was some evidence to suggest that women from lower socioeconomic backgrounds were more likely to drop out of the study. Future research should adopt a targeted approach to explore the effectiveness of the intervention for young women at higher risk of STIs.

Additional limitations were that we did not assess the researcher’s fidelity to the intervention delivered and the thematic analysis was conducted by only one researcher (the first author). Although there is evidence for the initial effectiveness of the intervention with regard to changing attitudes and increasing skill and self-efficacy, further work needs to be done to understand how this translates into actual condom use behaviour, especially with new or casual partners.

### Implications of findings

This feasibility study has demonstrated promising initial effectiveness and that young women are willing to experiment with condoms and lubes to keep themselves safe. In terms of cost, variety is the key requirement - providing young women with a choice of condoms and sachets of lubricant need not be expensive. In fact, the top-rated condom was not one of the ‘premium’ brands. It was rated highly because of the attractive, fun packaging, ease of opening and dots which provided extra stimulation. The appearance of condom packaging is something therefore that providers of free condoms to young women should consider.

Our findings also have implications for sex and relationships education in that providing a pleasure focussed approach is likely to be a more effective strategy to engage young women than traditional sex education. In terms of delivery, there is benefit in engaging external speakers with specialist knowledge. Again, this need not incur a high cost, as partnerships with local universities and colleges could provide this resource. In fact, some further education colleges had student sexual health ambassadors who provided peer-led education with regard to condom use.

## Conclusions

The objective of this study was to test the feasibility of a novel condom promotion intervention for young women. The intervention was implemented with no major adaptations required and the recruitment strategy proved effective. There was strong evidence for the acceptability of HERS-UK as young women engaged well with the intervention. There were no technical issues with Lifeguide, the web-based platform used for all online entries and text/email reminders were sent as programmed. HERS-UK is a viable and promising sexual health intervention for young women and a larger scale randomised controlled trial should be conducted to evaluate its effectiveness.

There was initial support for the potential effectiveness of HERS-UK on key outcomes and measures used were sensitive enough to capture change in outcome variables. There was a significant reduction in condom use errors and problems, improved attitudes and increase in self-efficacy for using condoms. The proportion of young women using condoms and lube for intercourse also increased from T1 to T2, and maintained at T3. However, motivation for using condoms did not change; assessment of motivation may require a different measurement approach, instead of asking participants to respond to one strongly worded statement. The overall mean at all time points, indicated a ‘neutral’ response to condom use. Even though attitudes improved, for some young women, sex would always feel better without condoms and motivation may be the most resistant to change. In fact, it is easier to correct condom use errors and problems — the ‘technical’ aspect, than it is to change actual condom use behaviour, which is why a ‘pleasure focus’ is so important.

For the majority of participants, the idea that condoms could be pleasure enhancing, or at least, not *interfere,* with sexual pleasure, was not something previously entertained. The information they had received about condoms in sex education sessions was very basic and clinical in the manner provided. Therefore, prior to participation in the study, most young women had not received any positive messages about condoms, other than that they protected against STIs and pregnancy. Negative attitudes are likely to be further entrenched by early problematic experiences and young women may never have the opportunity for those attitudes to be challenged.

The process interviews and open text comments highlighted the fact that many young women had also not fully considered their own pleasure and comfort during sex, being more concerned with their partner’s experience. The focus explicitly on female pleasure had provided the opportunity for young women to take time out and focus on themselves, which was very welcome, and is likely to be an effective component of any sexual health intervention for young women.

Future research needs to assess stakeholders’ perspectives on barriers and facilitators to implementing the HERS-UK intervention and also assess the crucial issue of cost-effectiveness of the intervention in a large-scale controlled trial involving a diverse sample and targeting young women most at risk of STIs.

## Supplementary Information

**Additional file 1.**

## Data Availability

Anonymised datasets used and/or analysed during the current study are available from the corresponding author on reasonable request.
