# Supplementary figures and images for: Feasibility study of the Home-based Exercises for Responsible Sex (HERS) intervention to promote correct and consistent condom use among young women
Source: Pilot Feasibility Stud. 2021 Jul 27;7:145. doi: 10.1186/s40814-021-00885-1 (PMC8314454; doi:10.1186/s40814-021-00885-1)

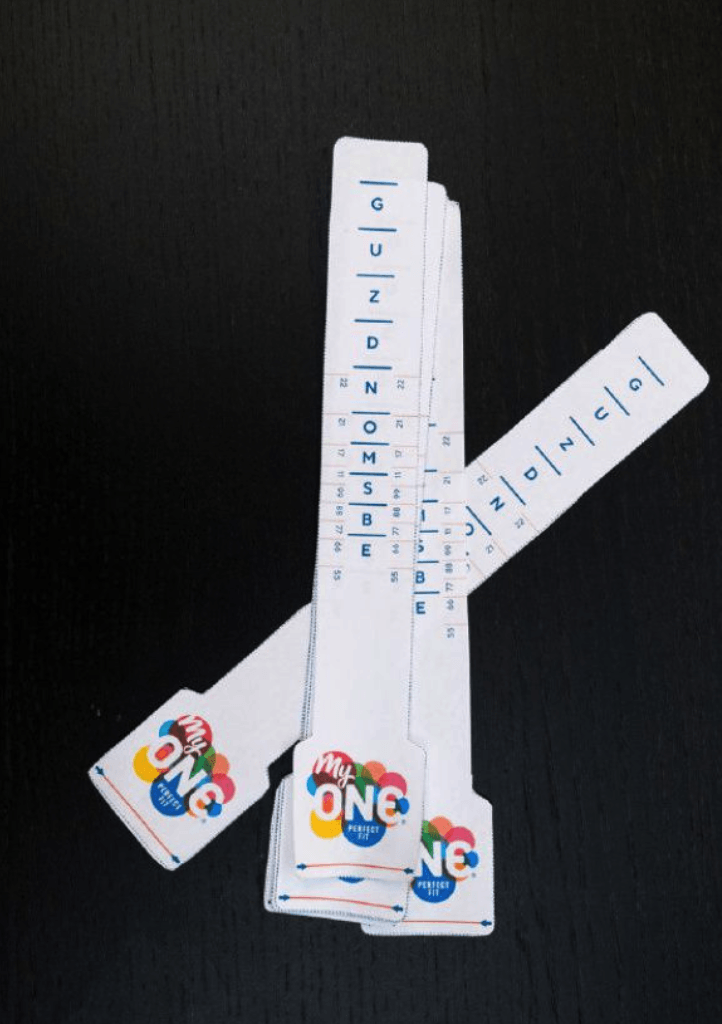

Supplement: Supplementary file 1 — Additional file 1. [file 40814_2021_885_MOESM1_ESM.zip › FITKITR2.png]

## Slide 1
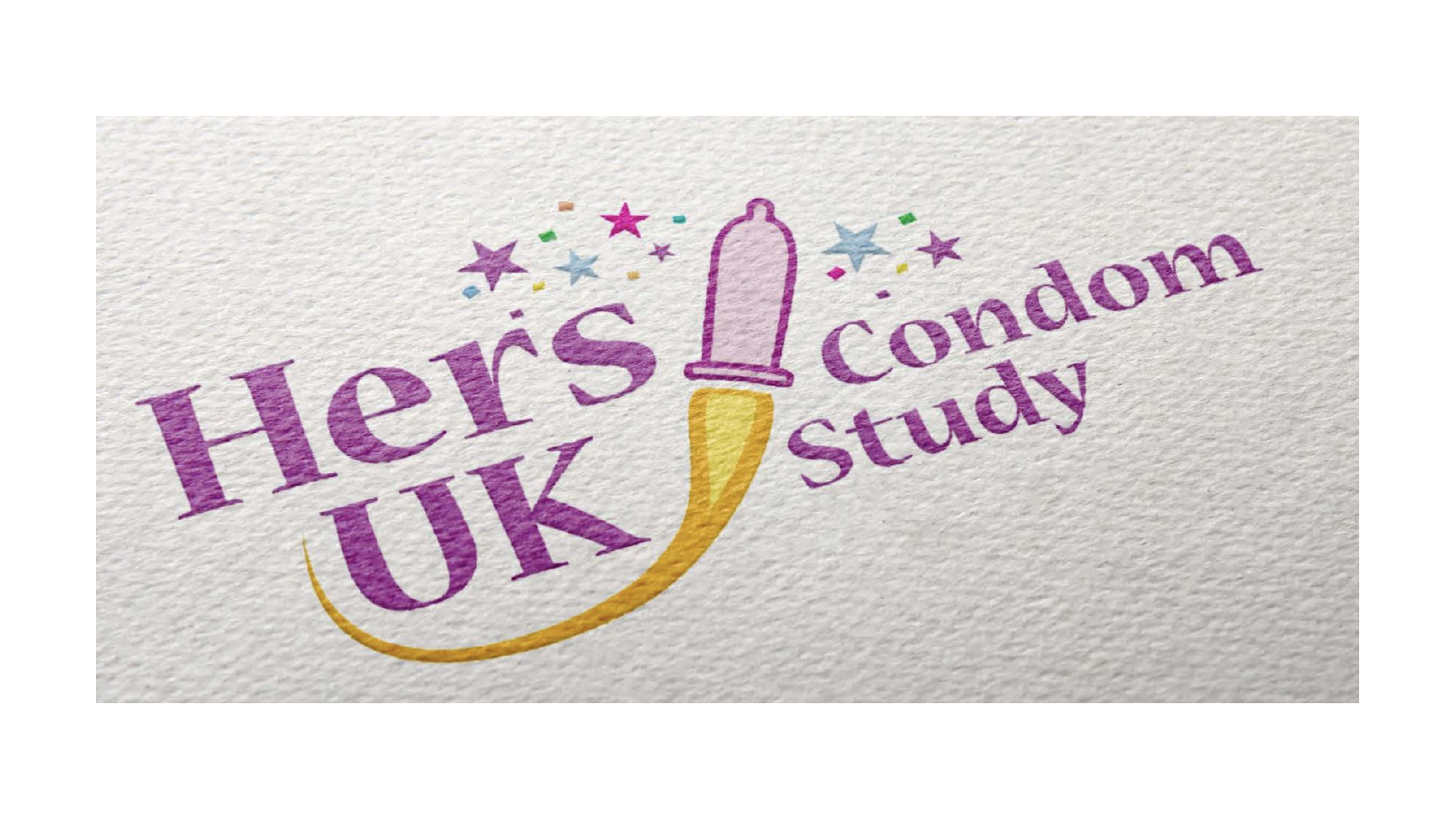

Supplement: Supplementary file 1 — Additional file 1. [file 40814_2021_885_MOESM1_ESM.zip › HERS study logoR2.pptx]

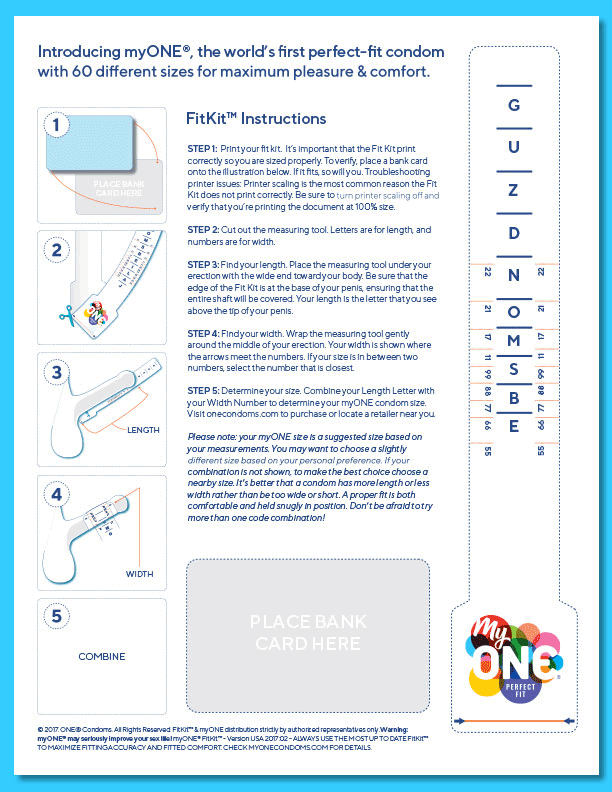

Supplement: Supplementary file 1 — Additional file 1. [file 40814_2021_885_MOESM1_ESM.zip › myOne-fitKitR2.png]
